# Autism Traits Predict Self-reported Executive Functioning Deficits in Everyday Life and an Aversion to Exercise

**DOI:** 10.1007/s10803-020-04741-8

**Published:** 2020-10-11

**Authors:** Lauren A. Mason, Brandon M. Zimiga, Regina Anders-Jefferson, Kenneth R. Paap

**Affiliations:** 1 San Francisco State University, San Francisco, CA USA; 2 Present Address: Department of Psychology, Tufts University, 490 Boston Ave., Medford, MA 02155 USA

**Keywords:** Autism traits, Autism quotient, Executive functioning, Exercise

## Abstract

Are Autism Quotient (AQ) scores related to executive functioning (EF)? We sampled 200 students of normal intelligence and examined the relationship between AQ scores and: (a) 5 self-ratings of EF, (b) 5 performance-based measures of EF, and (c) 5 types of activities or experiences that are assumed to recruit EF and sometimes enhance EF. Our findings reveal that as AQ scores increase, self-rated EF ability decreases. AQ scores and self-reported EF measures do not correlate with objective EF task performance. Furthermore, AQ scores were shown to be negatively associated with many specific types of physical activity. As AQ scores increase, individuals report fewer positive reasons for exercise and more rationalizations for not engaging in more exercise.

The diagnostic criteria for autism spectrum disorder (ASD) in the *Diagnostic and Statistical Manual of Mental Disorders* (American Psychiatric Association 2013) is comprised of two main categories. The first set of symptoms include deficits in: social–emotional reciprocity, nonverbal communication, and the development, maintenance, and understanding of relationships. The second set includes the presence of repetitive patterns of behavior, inflexible adherence to routines, and highly restricted and fixated interests. The DSM-5 diagnosis spans three levels of severity; this is consistent with the view that autism symptoms can be viewed as a cluster of behavioral tendencies that vary continuously along a spectrum.

## Autism and the Executive Dysfunction Hypothesis

Many of the categorical deficits observed in autism appear to be closely related to symptoms observed among individuals experiencing executive dysfunction. The concept of executive function (EF) has evolved over time and continues to do so. Luria (1966) has been credited for the idea of EF as a cognitive system responsible for intentionality, formulation of thoughts and actions, identification of goal-appropriate routines, and evaluation of outcomes. Further contributions to the literature categorized future-oriented processes, such as planning, as a part of EF (Denckla 1996). In more recent years, EF has been observed as a key, overarching component to the integration and management of more basic cognitive processes, such as sensation, attention, or memory (Eslinger 1996, 2008; Jurado and Rosselli 2007; Kodituwakku et al. 2001). In short, EF can be understood as a cognitive control system responsible for many mechanisms including the formulation of intentional thoughts and actions, the identification and achievement of goals, and the evaluation of outcomes via self-reflection.

The most obvious link between ASD and deficits in EF is that they share some of the same defining symptoms including difficulties switching attention and poor impulse control. They could have either shared or different etiologies. The possibility that they have a common cause, the executive dysfunction hypothesis, has been prominent for some time. Hill’s (2004) early and highly cited review concluded that autism is usually associated with deficits in mental flexibility and planning, but not inhibition.

A more nuanced question is whether ASD is related to general deficits in EF and whether specific EF tasks might be used as a broader autism phenotype. In a recent review, Demetriou et al. (2019) defined intermediate phenotypes for autism as markers associated with ASD that are heritable and present at higher rates within affected families than the general population. The possibility that EF may serve as a cognitive intermediate phenotype is particularly relevant within a framework like Miyake and Friedman’s (2012) unity and diversity model which assumes that EF consists of a set of latent factors (e.g., shifting and updating) that are separable, but still related to a higher-order ability because they are correlated with one another. This issue was addressed in a meta-analysis by Demetriou et al. (2018) that examined 235 comparisons between those diagnosed with autism and a control group across six domains of EF: concept formation, fluency, planning, response inhibition, switching/mental flexibility*,* and working memory. The overall effect size shows an EF deficit in those diagnosed of g = 0.6.^1^ The effect sizes across the six EF domains were remarkably similar. The most striking moderator was the method used to assess EF ability, as much larger effects (g = 1.8) were observed when EF was assessed in the form of self-ratings of behavioral tendencies in everyday life as compared to performance on laboratory tasks. This is interesting because it suggests that there is a distinction between self-reported and objective measures of EF ability. One purpose of this study is to investigate this relationship between self-reported autism traits and objective measures of EF.

Unsurprisingly, the deficits were somewhat smaller in adults than younger people. These findings, and our interest in college student populations led to the examination of Brady et al.’s (2015) study of EF deficits in young adults. The sample was comprised of 34 young adults diagnosed with ASD and 34 age- and sex-matched typically developing controls. The EF measures were drawn from the Delis–Kaplan Executive Function System (D-KEFS; Delis et al. 2001) which is a standardized set of nine tests that assess various components of EF. Some of the tests are specific instantiations of existing tools, such as measuring attention and inhibitory control by using a Stroop color–word interference task. The direct comparisons between the group means for the ASD and control groups showed statistically significant disadvantages in the Stroop measure of inhibition and the visual fluency measure of generativity.

While these findings clearly provide evidence for the hypothesis that ASD is associated with deficits in inhibition and generativity, the author’s conclusion is more nuanced: Brady et al. state, “…. these impairments are not substantial, on average, when compared with normative expectations” p. 7 and “….consequently appear to be spared in many individuals with HF-ASD” p. 8. A similar cautionary note was expressed by Geurts et al. (2014a, b): individuals with a clinical diagnosis of ASD have EF deficits more often than those without a diagnosis, but this entails that there are also individuals with autism who do not have any EF deficits.

In summary, the meta-analysis by Demetriou et al. (2018) showed small, but comparable deficits across six domains of EF. This pattern is consistent with the hypothesis that ASD and deficits in general EF tend to co-occur. However, work like that by Brady et al. and Geurts et al. revealed that individuals with autism often show deficits in only certain components of EF. Moreover, these individuals sometimes show no deficits when a “deficit” is defined as performance that would fall in the bottom 10% of normally functioning controls.

Given the inconsistencies in the literature, one primary purpose of our study is to further explore the relationship between EF and ASD. In doing so, we completely agree that EF accounts cannot be the sole explanation for ASD symptomology and that an individual differences approach is needed to make progress in understanding the etiology, prognosis, and treatment response for those with diagnosed ASD.

## Exploring the Relationship Between EF and ASD Using Traits Rather than Groups

### Avoiding Between-Group Designs

As described in the sections below, our research goals were quite broad. We aimed to expand the tests of deficits in the EF hypothesis to new or underutilized EF laboratory measures (a total of 5 measures based on task performance) and furthermore compared the association obtained with these objective tests to a set of 5 self-report measures of cognitive control in everyday life. Another major goal to be discussed later was to assess the relationship between ASD traits and participation in activities that are assumed to require or enhance EF by including a novel comprehensive exercise survey (CES). These goals pose a serious challenge to designing a typical study that compares a group diagnosed with ASD to at least one control group. For example, to detect a small effect size (d = 0.2) in a two-group design with a desired power of 0.8, the standard α of 0.05, and a one-tailed test, one needs 542 participants in each group. There are several advantages to exploring the relationship between ASD and many other factors by treating ASD as a continuum and avoiding the partitioning into groups. One tool that enables this approach is the autism quotient (AQ) scale described next.

### The Autism Quotient (AQ) Scale

The AQ is a brief, self-administered instrument for measuring the degree to which an adult with normal intelligence has traits associated with autism (Baron-Cohen et al. 2001). The scale provides a score of 0 to 50 across five behavioral subscales: attention to detail, deficits in social skill, attention switching, communication, and imagination. The developers view the AQ as a valuable instrument for rapidly quantifying where any given individual is situated on the continuum from autism to normality. Its utility rests on the assumption that autism lies on a continuum of social-communication disability and that effective treatments and interventions may derive from thinking of autism quantitatively rather than as a categorical diagnosis. As Gökçen et al. (2014) put it: “Theory and research suggests that features of autism are not restricted to individuals diagnosed with autism spectrum disorders, and that autism-like traits vary throughout the general population at lower severities” p. 187. However, see Gregory and Plaisted-Grant (2016) for an impassioned plea against accepting the continuum assumption uncritically.

Stevenson and Hart (2017) listed 50 studies that have identified and compared groups of high and low AQ individuals within neurotypical samples. Based on the authors’ conclusions, significant group differences were obtained across an array of research questions.^2^ Only five of these studies reported null results when group differences were predicted. Thus, AQ scores show a very strong propensity for revealing individual differences. Having said that, one should consider that published literature as a whole is biased to favor positive results against null results (Paap 2018; Paap et al. 2020b), and an unknown number of null results reside in researchers’ file drawers or have been rejected during peer review. Nevertheless, as expected from the symptoms of ASD, many of these differences show that high AQ individuals have deficits compared to those with low AQ (e.g., less accurate in recognizing anger, disgust, and sadness, Poljac et al. 2013). Note however that several findings highlight tasks where individuals with diagnosed autism and higher AQ scores actually tend to be advantaged (e.g., on embedded figures tests, Grinter et al. 2009; Almeida et al. 2010).

A major purpose of this study is to further test the executive dysfunction hypothesis by investigating associations between AQ scores and five performance-based EF measures. Mental flexibility was measured with switching costs and mixing costs measures derived from a color–shape switching task. Inhibitory control was measured as the interference score in a spatial Stroop task. The last two measures of EF were the slopes of target present and target absent trials on a conjunctive visual search task. The selection of the visual search task was motivated by recent emphasis on attention control as a better framework for understanding the inhibitory-control component of EF (Bialystok 2017; Burgoyne and Engle 2020).

## Performance Based Measures of EF Versus Self-ratings of Problems in Everyday Life

Recall that the Demetriou et al. (2018) meta-analysis on the relationship between ASD and EF showed larger effect sizes when EF was measured by subjective self-rating scales. As reviewed below, there is actually substantial evidence that performance-based measures of EF weakly correlate with self-report and may not measure the same construct. The possibility of multiple separate, but related constructs resonates with the variety of terms used to describe self-control challenges in everyday life: self-regulation, self-discipline, will-power, effortful control, ego strength, grit, and inhibitory control. Our interest in the relationship between objective measures of EF and self-control was originally piqued by Duckworth and Kern’s (2011) meta-analysis of 282 samples. Duckworth and Kern considered 12 types of performance-based EF tasks (e.g., Stroop, attention, set switching, etc.). The mean correlation between EF task performance and measures based on self-reports across the 12 EF domains tended to be quite low (ranging from r =  − 0.02 to + 0.18). At about the same time, McAuley et al. (2010) reported that another widely used self-rating questionnaire, the Brief Rating Inventory of Executive Function (BRIEF; Gioia et al. 2000), did not significantly correlate with performance-based measures computed from either the stop-signal (purported to measure response inhibition) or the N-back tasks (purported to measure working memory capacity).

More recent findings follow the same pattern. For example, Allom et al. (2016) showed near-zero correlations between three self-report measures of self-control and measures of EF derived from a stop-signal and Stroop color–word interference task. Paap et al. (2020a) targeted measures of interference control in four computer tasks (Simon, spatial Stroop, vertical Stroop, and flanker) and four measures of self-control based on self-ratings [Brief Self Control Scale (BSCS), Tangney et al. 2004; and the Premeditation, Urgency, and Perseverance scales of impulsivity, Whiteside and Lynam 2001]. Across the 16 combinations, the correlations between performance-based measures and self-reports ranged from − 0.075 to + 0.133. Although each of these correlations were based on more than 200 participants, none of them were statistically significant at p < 0.05. A reviewer of the original submission of this article pointed out that the results described above are also supported by Toplak et al. (2013). They reviewed 20 studies (13 using child participants) and reported that only 24% of 286 correlations were significant and that the overall median correlation was only + 0.19. Toplak et al. concluded that performance-based and rating measures of EF assess different levels of cognition: a conclusion that will be revisited in the discussion of our present findings.

One major purpose of this study is to continue to evaluate the relationship between performance-based measures of EF and self-report measures and to determine which construct has a stronger association with ASD. Thus, our design includes five measures of EF based on self-ratings: Tangney et al.’s (2004) BSCS, Whiteside and Lynam’s (2001) subscales of impulsive behavior, and Barkley’s (2011) Deficits in Executive Function Scale (BDEFS). Barkley’s (2011) Deficits in Executive Functioning Scale (BDEFS) is comprised of 89 questions split into five subdomains: Self-Management to Time, Self-Organization/Problem Solving, Self-Restraint/Inhibition, Self-Motivation, and Self-Regulation of Emotions. We expect to observe stronger relationships between autism traits and these self-rating measures of EF than in our laboratory task measures of EF ability.

## Activities Associated with the Development and Enhancement of EF

If ASD often involves deficits in EF, then this could have consequences for engaging in activities that are hypothesized to recruit EF and that might also enhance EF. For example, D’Souza et al. (2018) compared four groups defined by the combinations of bilingual or not and musician or not in multiple tasks tapping into EF. Results revealed that musically trained individuals, but not bilinguals, had enhanced working memory capacity (WMC), but neither skill enhanced inhibitory control as reflected in flanker or Stroop interference. When a difference emerges, like the one in WMC, there are multiple interpretations. Music performance may enhance WMC. Alternatively, those who already have superior WMC may enjoy more early success in music which, in turn, motivates additional training and practice. An individual with a deficit in EF (WMC in this example) is less likely to experience musical success and, as a result, performs and practices less. Furthermore, if music performance enhances general EF, this individual would be less likely to engage in an activity that would bolster their weakness.

In the present study, we tested for a relationship between autism traits and music performance, videogaming, bilingualism, and mindfulness meditation—an array of activities that have been hypothesized to recruit EF (see Paap et al. 2020a for a review). To the extent that ASD is associated with deficits in EF, then autism may be associated with lower levels of participation in these activities. Another activity potentially related to both autism and EF is exercise and other forms of physical activity. The role of exercise is discussed in the next section. This study focuses on physical activity specifically because research shows that as children with autism age, physical activity engagement decreases (MacDonald et al. 2011). While there is a general consensus that exercise is essential to good health, the evidence suggesting that autism is associated with an aversion to physical activity highlights the need for further research on the underpinnings of this association.

## Exercise, EF, and Autism

Not surprising, it is commonly assumed that exercise improves physical fitness. Likewise, there is a consensus that EF predicts achievement, health, wealth, and quality of life (Moffitt et al. 2011). Other relationships are settled to lesser extents. Does exercise enhance EF? If yes, how does mode, frequency, duration, and intensity moderate the relationship? Drawing on their own meta-analysis, one of Diamond and Ling’s (2016) conclusions was that without a cognitive component, aerobic exercise or resistance training produces little or no EF benefit. Stated more poetically, EF benefits from mindful, but not mindless physical activity. Hillman et al. (2019) respectfully, but forcibly, disagreed with the mindful, but not mindless conclusion as they believed Diamond and Ling’s meta-analysis failed to consider all of the relevant articles, that the results of some were misinterpreted, and that some of the interventions were mischaracterized. Part of the evidence reviewed by Hillman et al. supporting the benefits of exercise was a recent meta-analysis by Northey et al. (2018). The analyses showed greater benefits when the frequency of exercise was high (5–7 times per week), of at least medium duration (45 to 60 min), and of at least moderate intensity.

Exercise can also reduce adverse symptomology of ASD such as stereotypy, aggression, off-task behavior, and elopement (Lang et al. 2010). While research reflects that those with autism are less likely to engage in exercise, the reason for this relationship is not completely understood. It is the case that more than 50% of children with ASD have movement deficits (Green et al. 2009) and poor motor coordination or balance that limits their ability to be successful in some physical activities (Potvin et al. 2013).

Our study seeks to provide further understanding of the link between autism traits, EF, and exercise. It is important to include both types of EF measures (self-report and laboratory-based) in the design as Allom et al. (2016) reported a significant and positive correlation between a composite measure of trait self-control and self-reported physical activity, but correlations near zero for performance on either a stop-signal or a Stroop task.

## Research Question

In the present study, a sample of 200 university students completed the AQ scale of autism traits, 5 measures of EF in everyday life based on self-ratings, 3 laboratory tasks of cognitive ability, a comprehensive survey of exercise and physical activity, and a general background survey on various demographics including the number of languages spoken and the frequency of music performance, videogaming, and mindfulness meditation. The relationships between AQ, EF (both self-reported based on behavioral tendencies in everyday life and performance on laboratory tasks), and the frequency of engaging in life activities related to EF (e.g., exercise) were explored. If individuals do occupy locations on a continuum of autism traits, then one line of expectation is that students who have higher AQ scores will do poorer on tests of EF (with the possible exception of visual search), engage less frequently in activities that require EF, and exercise less often. Given the significant negative correlations expected and obtained between AQ and physical activity, we were also able to probe the reasons why participants engage in the physical activities that they do regularly and the reasons that prevent them from engaging more.

## Method

### Participants

The sample consisted of 200 students between the ages of 18 and 45 with a median of 19. Participation either earned extra credit or fulfilled a course requirement. The university sample is diverse with the largest groups of students in the study identifying as Hispanic (29%), Asian (28%), and White (21%). 57% Identify as female. A focus on university students is worthwhile; as the number of children identified with an autism spectrum disorder increases, the fastest growing sub-group is those without co-occurring intellectual disability (Baio 2012). Furthermore, as highlighted in the Special Issue Preface on College Experiences for Students With Autism Spectrum Disorder, due to improved treatment practices, increased numbers of students with ASD are seeking a college education, with 45% of the 550,000 children with ASD in the USA expected to enroll in a post-secondary education in the future (Reichow and Volkmar 2011; Volkmar et al. 2017; Buescher et al. 2014; Newman et al. 2011; Jackson et al. 2018). Yet, these students experience unique challenges which the literature sparsely addresses. This moreover exemplifies the necessity to focus increased attention on how autism traits are characterized in college samples.

### Materials and Procedure

The study was conducted in a single session of approximately 90 min. At the start of the session, informed consent was obtained for a protocol approved by the sponsoring University’s Institutional Review Board. All subjects completed the following tasks: (1) color–shape switching, (2) spatial Stroop, (3) visual search, (4) Raven’s, (5) AQ, (6) BDEFS, (7) BSC, (8) Premeditation, (9) Urgency, (10) Perseverance, (11) Comprehensive Exercise Survey (CES), and (12) a background questionnaire covering language history, demographics, frequency of engaging in music, videogaming, and mindfulness.

#### Color–Shape Switching

The color–shape switching task is commonly used to derive switching costs and mixing costs that typically serve as measures of the shifting and monitoring components of EF, respectively. Both a shape and a color task are used. Each trial in the mixed block began with a fixation cross for 350 ms. Then, a cue was presented for 250 ms that specified the required response: a rainbow for a decision based on color and a superimposed triangle/circle for a shape decision. The cue was immediately followed by one of the four possible targets: red circle, red triangle, green circle, or green triangle. In the shape task, the participant was directed to respond to the shapes while ignoring the color. In contrast, in the color task, the participant was told to respond to the color while disregarding the shape. Two fingers of one hand were used to make color decisions, while two fingers of the other hand were used to make shape decisions. Half of the trials require a task switch from color to shape, or vise versa, and half are repeated trials which do not require a task switch. The difference between repeat trials and switch trials in the mixed block indicates switching costs. Two single tasks are included each consisting of 36 pure color and 36 pure shape trials. The mixed task with task cues contains 3 blocks of 48 trials. The difference between repeat trials in the mixed block and the mean of the pure blocks indicates mixing costs.

This exact instantiation of the color–shape switching task shows test–retest reliability over a 1-week interval of r = 0.62 for switching cost and r = 0.75 for mixing costs (Paap and Sawi 2016). By general standards, these are less than desirable, but as Paap and Sawi point out, these reliabilities are as good or better than those typically observed for dependent measures based on RT difference scores. Furthermore, the switching-cost measure derived from this exact color–shape task demonstrated convergent validity because it formed a coherent latent variable with two other cued switching tasks (Paap et al. 2017).

Geurts et al. (2009) reviewed the “paradox” of cognitive flexibility in autism. They observe that both clinicians and researchers widely believe that cognitive flexibility deficits are pathognomonic of ASDs; however, their review of five categories of tasks used to measure flexibility led them to conclude that there was no consistent evidence of deficits. They further considered the extent to which the inconsistencies are due to measurement problems or to the heterogeneity of the autism spectrum given that there are substantial individual differences in the type of difficulties individuals with autism experience. Geurts et al. present an insightful discussion of the task-impurity problem in the Wisconsin Card Sorting Task (WCST) and Intra Dimensional–Extra Dimensional (ID–ED) that eventually leads to their endorsement of task-switching paradigms such as the color–shape switching task: “This more mechanistic approach probably is the best way to study cognitive flexibility” p. 7. Geurts et al. share that only four studies investigated task-switching in individuals with autism and none of them reported performance deficits. This is true, but the implications of the null results may be limited because they were based on small ASD groups (n’s range from 10 to 23) and furthermore substantially modified the standard task-switching paradigm that is exemplified by the color–shape switching task described in the previous paragraph.

#### Spatial Stroop

Each trial began with the presentation of a center fixation (+) for 500 ms. The center fixation was immediately followed by the target stimulus which was either a “>” or a “<”. In this task, participants are instructed to press the left key if the arrow points to the left, and the right key if the arrow points to the right. The participants were instructed to respond as quickly as possible without making errors. In a neutral block the target was displayed either 2.3° above or below the center fixation. In the spatial Stroop block the target was displayed either 3.9° to the left or to the right of the center fixation. A trial was defined as congruent if the location of the target was on the same side as the correct response and as incongruent if the location of the target was on the opposite side. The standard marker of inhibitory control within this task is the difference in mean response times between congruent and incongruent trials. All participants started with two neutral blocks of 20 trials. The first block was considered practice. The neutral blocks were followed by two spatial Stroop blocks. Each of these consisted of 20 congruent and 20 incongruent trials presented in random order. The test–retest reliability of a similar spatial Stroop task was r = 0.71 for 17 normal adults tested over a period of about 11 weeks (Wöstmann et al. 2013).

The spatial Stroop task is superior to the original Stroop color–word when the groups being compared may differ in receptive language ability as the interference generated from the predisposition to read the word increases with reading skill and automaticity. For example, Adams and Jarrold (2009) showed that children with autism were actually better than matched controls on the color–word Stroop but performed equivalently in two tasks where the to-be-ignored and incongruent task was not reading words. In the only comparison between ASD and matched controls using the spatial Stroop task, there were no group differences (Schmitz et al. 2006), although there were concurrent group differences in regional fMRI brain activation. As described in the introduction, inhibitory control measures are somewhat notorious for lacking convergent validity. Yet, Paap et al. (2019) used an exploratory factor analysis to show that the spatial Stroop and two versions of the Simon task formed a coherent latent variable that excluded the flanker task. Despite the null results reported by Schmitz et al. (2006) and the measurement problems, a theoretical framework that assumes that ASD is associated with deficits in inhibitory control would predict that interference scores will increase with AQ scores.

#### Visual Search

Only the low discriminability conjunctive search condition from Friesen et al. (2014) was used in the current study. The target stimulus was a blue triangle. The search was conjunctive because the distractors included purple triangles and blue diamonds. The search involved low discriminability because blue is similar to purple and diamonds are similar to triangles. The target appeared randomly in 1 of 26 designated locations on the screen. There were 24 trials in which the target was present. These were equally divided between distractor set sizes of 0, 5, 15 or 25 distractors. The 18 target-absent trials were equally divided between 5, 15, and 25 distractors. Participants were instructed to press the “1” key the target was present and press the “0” key if it was not. After the response, the next array was presented.

Our conjunctive visual search task was quite similar to the ones used by Plaisted et al. (1998) and O’Riordan et al. (2001). Both of these studies showed that children (mean = 8 years) with a diagnosis of autism performed better than normally developing controls, especially on the more difficult target-absent trials. The classic interpretation of conjunctive search, Treisman and Gelade’s (1980) feature-integration theory of attention, renders this ASD advantage in conjunctive search surprising. The theory assumes that focused attention is required in order to conjoin features like shape and color into a single object. If ASD entails attention to detail and a preference for features over integrated wholes, then the ASD group should find conjunctive search difficult.

#### Raven’s Progressive Matrices

A potential confound with fluid intelligence was assessed using Set 1 of the Raven’s Advanced Progressive Matrices (Raven et al. 1977). The Raven’s task was computerized and consisted of 12 items total; participants were given a maximum of 2 min to select from a set of 8 possible choices for each puzzle, each consisting of a pattern with a piece missing. Fluid intelligence is assessed using Set 1 of the Ravens Advanced Progressive Matrices (Raven et al. 1977). Participants are told to “Look at the pattern, think what the missing part must be like to complete the pattern correctly.” Participants are provided with 8 options to choose from and are allotted a maximum of 2 min to respond to each item.

#### Autism Quotient (AQ)

Traits associated with autism are measured using the AQ scale (Baron-Cohen et al. 2001). This measure has been described as a valuable instrument for rapidly quantifying where an individual is situated along the continuum from autism to normality. The AQ consists of 50 questions with four scaled-response options ranging from “definitely agree” to “definitely disagree.” For example, item 1. states, “I prefer to do things with others rather than on my own.” Baron-Cohen et al. introduced a binary scoring rubric whereby one point was awarded if the participant indicated either definite or slight agreement with an autism trait and otherwise zero. Thus, possible scores ranged from 0 to 50. For a group of 58 adults with Asperger syndrome (AS) or high-functioning autism (HFA) with mean age of 32 years the mean AQ score was 35.8 compared to 16.4 for a group of matched controls, and 17.6 for a group of university students. There was a small amount of overlap at the high end of the distributions. For example, whereas 79.3% of the AS/HFA group scored 32 or more, only 2.3% of the control group did so. However, 6% of a sample of Cambridge University students scored 32 or higher. Baron-Cohen et al. conclude that “A score of 32+ appears to be a useful cut-off for distinguishing individuals who have clinically significant levels of autistic traits” p. 15. There was very little overlap at the low end as 40.6% of the controls had AQ scores less than 18, but no one from the AS/HFA group did so. Control males scored higher than control females, but there were no sex differences in the AS/HFA group or in the group of university students. The test–retest reliability for a group of 17 university students was r = 0.7.

#### Barkley Deficits in Executive Functioning Scale (BDEFS)

The BDEFS is comprised of 89 questions split into five subdomains: Time Management, Organization/Problem Solving, Self-Restraint/Inhibition, Self-Motivation, and Self-Regulation of Emotions (Barkley 2011). Items are presented as “problems” and participants are asked to circle the response that best describes their behavior, that is, how often they experience this problem during the past 6 months: never or rarely, sometimes, often, or very often. Barkley equates attention deficit hyperactivity disorder (ADHD) with deficits in EF and hence with performance on the BDEFS. If BDEFS and AQ are strongly correlated this would indicate that the disorders are related (with respect to these subjective measures) and that deficits in EF are prevalent in ASD.

The internal consistency, measured as Cronbach’s α, of each of the subscales is satisfactory ranging from 0.91 to 0.96 with a median of 0.95. Based on a sample of 62 normal adults the test–retest reliability for the overall BDEFS score was r = 0.84 (Barkley 2011). The test–retest reliability for the five subscales ranged from r = 0.62 to 0.90 with a median of 0.78.

#### Trait Self-control

Subjective measures of personal self-control were obtained using the BSCS (Tangney et al. 2004). This 13-item questionnaire is widely used throughout the literature on self-control and has been proposed as a predictor of both beneficial and adverse outcomes. Higher levels of personal self-control are indicated by higher scores on the scale. The BSCS and BDEFS should strongly correlate to the extent that self-control and executive functioning are the same construct. The internal reliability of the BSCS is good with αs of 0.83 and 0.85 in the two studies reported by Tangney et al. (2004). Likewise, the test–retest reliability for the BSCS was good (r = 0.87) over a three-week delay for a sample of 233 university students.

#### Trait Impulsivity

Three of the UPPS Impulsive-Behavior subscales developed by Whiteside and Lynam (2001) were included: (lack of) premeditation, urgency, and (lack of) perseverance. Their fourth facet, sensation seeking, was not included because it showed the weakest correlations with the other three facets (Whiteside and Lyman 2001) and with a variety of measures of EF (Duckworth and Kern 2011). Whiteside and Lyman (2001) interpreted their four distinct factors as “discrete psychological processes that lead to impulsive-like behaviors” (p. 685). The α reliabilities were 0.87, 0.89, and 0.83 for (lack of) Premeditation, Urgency, and (lack of) Perseverance, respectively. Weafer et al. (2013) reported test–retest reliabilities for the UPPS based on a sample of 126 healthy adults showed correlations ranging from r = 0.81 to 0.86 across the subscales. Paap et al. (2020a) reported significant correlations between the BSCS and the trait impulsivity scales.

#### Comprehensive Exercise Survey (CES)

Because a pilot study (Mason et al. 2018) showed significant correlations between AQ scores and the two items described below, a comprehensive exercise survey (CES) was developed and included in the present study that looked at the frequency, duration, and intensity of various types of exercise and physical activity and the reasons for engaging in the types of exercise an individual does, as well as reasons that prevented them from doing more. The exercise item used in earlier work read: “How often in a typical week do you exercise, work out, or participate in a sport?” Responses were based on a 5-point Likert scale ranging from *never* to *very often* and the correlation with AQ scores was r(129) =  − 0.36, p < 0.001. A related, but distinct item emphasized ability, not frequency of participation: “Team sports often involve dividing your attention between a ball, a goal, your opponents, and your teammates. Do you excel at these sports?” Responses were based on a 5-point Likert scale ranging from *No, I am far below average* to *I am much better than average.* The correlation between this item and AQ scores was r(129) =  − 0.18, p = 0.046.

The CES assesses activities in this order: (1) walking, (2) climbing stairs, (3) single-person exercise, (4) team sports, (5) one- or two-person ball sports, (6) contact/combat sports, (7) dance, (8) other activities not probed, (9) physical activity at home, and (10) physical activity at work. For Categories 1 to 8 frequency was assessed with an item like this: *How often do you bike?* And a 9-point Likert scale: *never or rarely, a few times per year* [0]*, 1 or 2 times a month* [.33]*, 1 time per week* [1]*, 2 times per week* [2]*, 3 times per week* [3]*, 4 to 5 times per week* [4.5]*, 6 to 7 times per week* [6.5]*, multiple times per day* [14]*.* The response selected was recoded to frequency per week as shown in parentheses in the previous sentence. If the participant indicated any frequency greater than zero, they were then asked to report the typical duration per session. The range of responses was adjusted for the different activities and sports. For example, the choices for biking were 15, 30, 45, 60, 75, 90, 105, 120, 150 min per session. A Total Time per week for each type of exercise/activity was computed by taking the product of frequency and time, for example, 3 times a week × 30 min per session = 90 min per week. A higher order composite, Energy, was calculated by multiplying Total Time by the selected Intensity Level for each activity/sport. The intensity probe read *What is your average level of effort (intensity) for each activity? Low: small increases in breathing or heart rate, Moderate: noticeable increases in breathing, heart rate, and perspiration, High: considerable increases in breathing, heart rate, and perspiration.* The three levels were coded as 1, 2, and 3. For a competitive cyclist this may yield a Total Energy score of 7 (days per week) × 105 (min per session) × 3 (high intensity) = 2205. In addition to biking, the single-person exercise category included climbing (rock/mountain/wall), exercising (with weights, machines, or floor), skateboarding (skating), running, surfing, swimming, and yoga (tai chi, pilates). Although we refer to these activities as “single person” they can, of course, be done either alone or with others. Thus, in a following item we ask if they prefer to do them alone or with others.

The team sports include basketball, baseball (softball, cricket), hockey (ice, inline, field, lacrosse), football (American), rugby, soccer, volleyball, and water polo. The one- or two-person ball sports included bowling, golfing, handball (racquetball, squash), table tennis (ping pong), tennis (badminton). The contact/combat sports were boxing, martial arts, and wrestling. *How often do you dance?* was treated as a separate category. To reiterate, for each activity that the participant reported the participant was asked to indicate the frequency, duration per session, and intensity. The product of frequency and duration was used to calculate Total Time per week. Energy per week was computed as the sum of the products of Total Time and Intensity for each activity.

A somewhat different approach was taken to measure physical activity at home and at work. For example, we asked: *On a typical weekday, how many hours are you at home and not sleeping.* This was followed by: *What percentage of the time when you are at home and awake is spent in activities at each of the indicated levels of effort? Sitting (watching TV, using computer/phone, reading, homework, conversation, etc.), Standing (at sink/basin, at standing desk, at work bench, ironing, etc.), Moving about with lifting, carrying, pushing, bending, or squatting.* Based on the amount of time spent at home awake per weekday and the percentage of time spent in each mode, the total amount of time spent sitting, standing, and moving can be computed for each individual and combined with similar estimates for probes about the weekend and time spent at work. Finally, sitting, standing, and moving were treated as three intensity levels and assigned the weight of 1.0, 1.5., and 2, respectively. Thus, the total time in each mode (sitting, standing, and moving) can be summed for weekdays at home, weekends at home, and time spent at work. Total energy per week at home and at work can also be computed by multiplying the total times by the intensity weights.

Total time and energy expended per week walking were built up from separate probes gathering information about walking while commuting to the university, walking while commuting to work, and walking a dog or walking (hiking) for pleasure. Stair walking was assessed with this item: *How many flights of stairs do you walk up on a typical day? None* [0]*, 1 to 5* [3]*, 6 to 10* [8], *11 to 15* [13]*, 16 to 20* [18]*, more than 20* [25]*.* The number of flights was coded as indicated by the square brackets for each Likert value. We used 30 s per flight of stairs to estimate the total time climbing stairs per week. As usual, the amount of energy was computed by multiplying total time by the reported intensity level for stair climbing. Finally, because each type of physical activity or exercise yielded an estimate of Total Time and Energy, Per Week they can be summed to estimate the Grand Total Time of physical activity and the Grand Energy Expenditure per week.

#### Background Questionnaire

Participants were asked to respond to a number of demographic questions pertaining to their background, including the educational level obtained by their parents. They were also asked to rate their frequency of participation in activities such as video games, musical instruments, exercise including team sports, and meditation/mindfulness, in the form of a single question for each activity.

## Results

### AQ Descriptive Statistics

#### Binary Scoring

Using the binary scoring method introduced by Baron-Cohen et al. (2001) our usable sample of 200 students had a mean of 19.9 (SD = 5.3). This is slightly greater than the means reported by Baron-Cohen et al.: (a) for their control group (M = 16.4), (b) for their group of Cambridge students (M = 17.6), and by Ruzich et al. (2015) in a review of 73 published studies (M = 16.9). Two point 5% of our distribution scored 32 or above, the cutoff Baron-Cohen suggested “….for distinguishing individuals who have clinically significant levels of autistic traits.” The 2.5% is very similar to the proportion obtained in Baron-Cohen et al.’s control group (2%), but somewhat less than the 6% reported for Cambridge students. In general, our distribution of AQ scores derived from the binary scoring method appears to be reasonably close to that obtained by the scale developers.

#### Likert Scoring

By ignoring the distinction between definite and slight agreement (or between disagreement and slight disagreement) the binary scoring method potentially discards useful information. Austin (2005) proposed to retain that information and use the Likert values of 1, 2, 3, and 4 to score the responses *definitely disagree*, *slightly disagree*, *slightly agree*, and *definitely agree*, with reverse scoring as applicable. Note that the larger numbers are assigned to the “agree” responses and that, consequently, larger scores indicate greater agreement with autism traits. In a direct comparison of the two methods Stevenson and Hart (2017) reported that Likert scoring yielded higher internal consistency and test–retest reliability for both the overall AQ scores and for the individual subscales. For example, the test–retest reliability for the overall scores and for a sample of 178 university students was r = 0.82 for binary scoring and r = 0.86 for Likert scoring. Likewise, the correlations reported below were always stronger using Likert scoring. We report mean Likert scores (not the total of all 50 items) such that the possible scores range from 1.0 to 4.0 with an average rating of 2.5 representing the neutral point.^3^

#### The Distribution of AQ Likert Scores

**Fig. 1:**
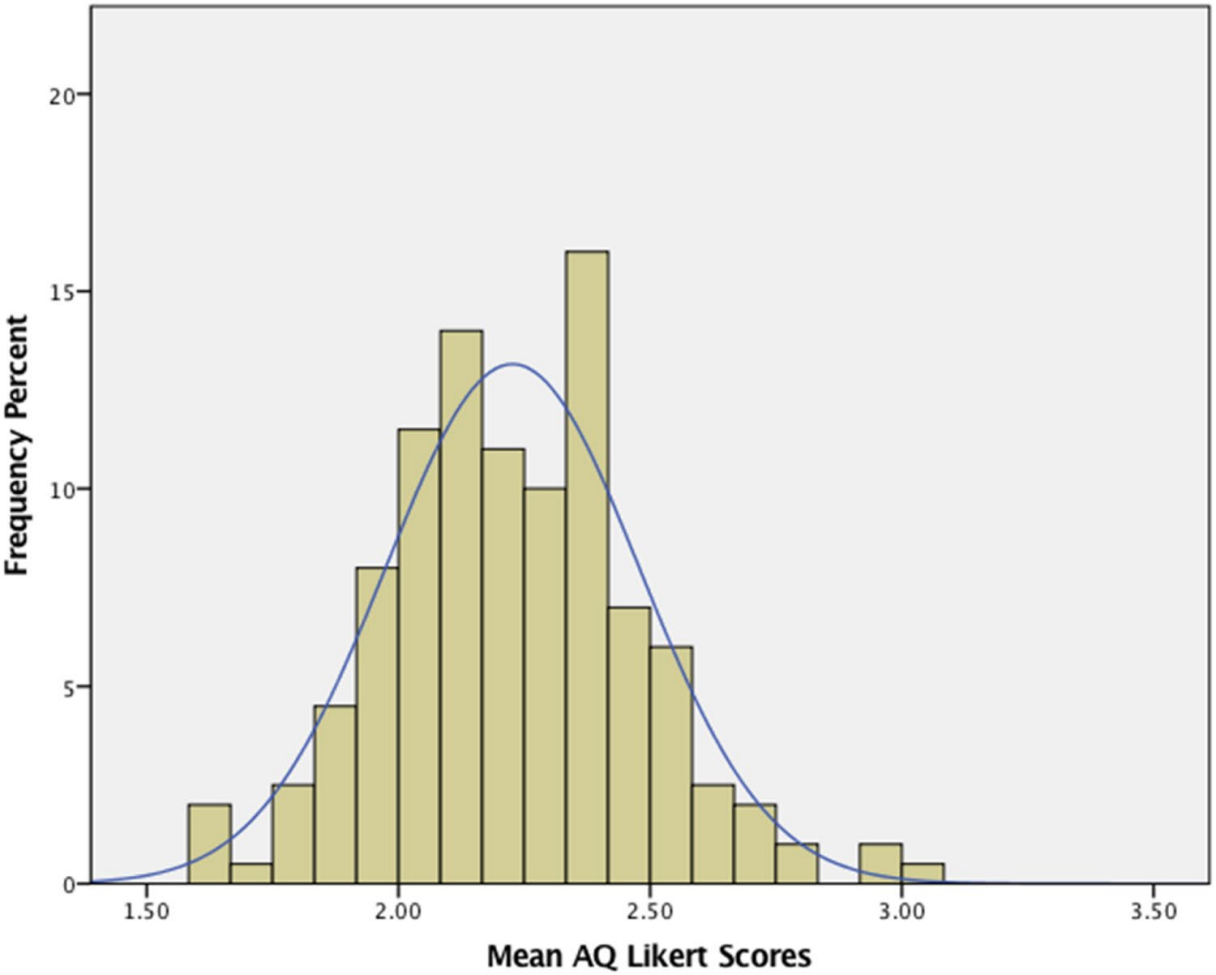
Distribution of AQ Likert scores for the sample of 200 students

There is no principled way to partition the continuum of AQ scores into subgroups (see Stevenson and Hart 2017, for an excellent discussion) and partitioning a continuum into subcategories can result in the loss of information. Consequently, most of the analyses reported below use AQ scores as a predictor or outcome variable in a correlational analysis. Thus, it is important to show that the distribution of AQ scores has desirable properties. As shown in the distribution of AQ scores (Fig. 1) and in the Kolmogorov–Smirnov tests (Table 1) the AQ scores do not depart from normality and do not have marked skewness or kurtosis.

**Table 1:** Descriptive statistics for AQ Likert and laboratory measures of EF

	AQ Likert	Spatial Stroop	Switch cost	Mixing cost	Target present	Target absent
N	200	198	197	196	200	200
Mean	2.22	42	189	301	26	50
SD	.25	35	107	144	15	25
Skewness	.23	.84	.49	1.23	.67	.51
SD of skewness	.17	.17	.17	.17	.17	.17
Kurtosis	.47	2.31	.49	2.02	1.64	.21
SD of kurtosis	.34	.34	.34	.35	.34	.34
Min	1.6	− 80	− 109	52	− 24	− 13
Max	3.1	217	508	945	90	137
K–S statistic	.05	.07	.07	.10	.07	.07
K–S sig	.20	.02	.03	.00	.01	.01

*K-S* Kolmogorov–Smirnov

Our mean of 2.22 is very close to the mean of 2.18 reported by Stevenson and Hart (2017) for 403 undergraduate students (mean age = 19.3 years). Likewise, our mean is nearly identical to the average of the 13 sample means (2.18) reviewed by Stevenson and Hart. Our sample appears to be typical for neurotypical young adults.

#### Sex, Age, SES, and Fluid Intelligence

Based on self-reports of sex-at-birth our student sample consisted of 31% males and 69% females. Thus, males were underrepresented, even relative to the university enrollment (43%). The mean Likert AQ scores for females (2.25) and males (2.18) did not significantly differ, *t*(197) = 1.84, *p* = 0.067. The absence of a sex effect replicates the null result reported by Stevenson and Hart (2017). However, AQ scores did decrease with age, r(199) =  − 0.204, p = 0.004. Neither the composite (father’s education, mother’s education, family income) measure of SES nor the Raven’s test of fluid intelligence were significantly correlated with AQ scores: *r*(199) =  − 0.02, *p* = 0.750 and *r*(198) =  + 0.08, *p* = 0.24, respectively.

### Performance-Based Measures of EF

Each participant completed three laboratory tasks commonly used to derive measures of EF: spatial Stroop, color–shape switching, and conjunctive visual search.

#### Spatial Stroop Task

The RT differences between congruent and incongruent trials are referred to as interference scores or Stroop effects and typically used as a measure of selective attention or inhibitory control. The mean Stroop effect of 42 ms was significant, *t*(197) = 12.11, *p* < 0.001. For young adults, this is a typical and robust interference effect. Overall accuracy was 94.0% correct.

#### Color–Shape Switching Task

The RT differences between repeat and switch trials from the mixed block are referred to as switch costs and are typically used as a measure of switching or shifting ability. The mean switch costs of 189 ms was significant, *t*(197) = 24.91, *p* < 0.001. Overall accuracy was 92% correct. The RT differences between the single-task blocks and the repeat trials from the mixed block are referred to as mixing costs and are typically used as a measure of the monitoring or updating component of EF. The mean mixing costs of 301 ms was significant, t(195) = 29.18, p < 0.001. Overall accuracy was 96% correct. For young adults both the switching costs and mixing costs were typical and robust.

#### Conjunctive Visual Search

In conjunctive visual search, search time is typically a linear function of the number of distractors with slopes on target present trials typically one-half of that on target absent trials. This follows from the assumption that search is serial and self-terminating and that the target is randomly located within the display. The sample means conform to these norms with a slope of 25 ms/item on the target present trials and 51 ms/item on target absent trials. Conjunctive visual search is not typically used as a measure of EF, but Friesen et al. (2014) assumed that it reflected a measure of the ability to disengage attention.

### Correlations Between Likert AQ Scores and Performance-Based Measures of EF

The correlations between AQ scores and each of the five objective measures of EF were non-significant at the conventional α level of 0.05. Those bivariate correlations are shown in Table 2. With this said, the largest correlation is with target-absent slope in the visual search task, *r*(197) =  − 0.131, *p* = 0.067, where smaller slopes are associated with greater AQ scores. As smaller slopes signal faster and more efficient visual search times, better performance is associated with greater autism tendencies. Although this is a small effect (and non-significant at the convention α of p = 0.05), it is consistent with the results reported and reviewed by Keehn and Joseph (2016) showing that children and adolescents with autism spectrum disorder (ASD) often show superior visual search performance on target absent trials. It is also consistent with Almeida et al. (2010) who reported superior performance for a high AQ group of undergraduate students in a visual search task that involved embedded figures in two of the three conditions. Almeida et al. favored an interpretation based on weak central coherence (WCC) theory (Frith 2003) which proposes that superior performance by individuals with autism is related to a bias toward perceiving the simple elements at the expense of combining them to perceive the integrated “whole”. As discussed earlier, a bias to attend to simple elements over the whole does not logically predict an advantage in conjunctive visual search.

**Table 2:** Bivariate correlations between AQ Likert scores and laboratory measures of EF

	Spatial Stroop	Switch cost RT	Mixing cost RT	Target present slope	Target absent slope
Pearson r	+ .102	− .131	+ .034	− .003	− .131
Sig. (2-tailed)	.15	.61	.64	.97	.07
N	195	194	193	197	197

### Correlations Among the Performance-Based Measures of EF

EF is often viewed as an umbrella construct that consists of a number of related components such as shifting (switching), updating (working memory capacity), and inhibition (Miyake and Freidman 2012). Within this framework, measures of the same component should be highly correlated and measures loading on different components weakly correlated. The correlations between the five EF measures are shown in Table 3. The only significant cross-task correlation is between the spatial Stroop effect and switch cost, *r*(194) = 0.184, *p* = 0.01.

**Table 3:** Bivariate correlations between the lab measures of EF

	Switch cost RT	Mixing cost RT	Target present slope	Target absent slope
Spatial Stroop	+ .184*	+ .101	+ .020	− .079
Switch cost RT		+ .175*	+ .083	− .079
Mixing cost RT			+ .060	+ .178*
Target present slope				+ .429**

### Self-rated Measures of EF in Everyday Life

#### Barkley Deficits in Executive Functioning Scale

BDEFS items are prompted with this instruction: *How often do you experience each of these problems?* For example, *Procrastinate or put off doing things until the last moment.* Responses are a Likert scale consisting of *1 Never or rarely, 2 Sometimes, 3 Often, and 4 Very often.* The mean of the 89 items for each participant was computed, with reverse scoring as applicable. Thus, item scores range from 1 to 4 with a neutral point of 2.5. Characteristics of the distribution of BDEFS scores are shown in Table 4. The mean of 1.9 is less than the neutral point and it is important to remember that this is a scale of “deficits” in EF and that higher BDEFS scores reflect less EF ability.

**Table 4:** Descriptive statistics for self-report measures of EF

	BDEFS	BSCS	Premeditation	Urgency	Perseverance
N	200	200	200	200	200
Mean	1.9	40.2	1.9	2.3	2.1
SD	.44	9.4	.52	.64	.55
Skewness	.52	.18	.68	.25	− .30
SD of skewness	.17	.17	.17	.17	.17
Kurtosis	.19	− .30	1.27	− .50	− .00
SD of kurtosis	.34	.34	.34	.34	.34
Min	1.03	14	1.0	1.00	1.0
Max	3.43	65	4.0	4.00	3.7
K–S statistic	.07	.04	.08	.08	.07
K–S sig	.01	.20	.004	.004	.01

#### Tangney, Baumeister, and Boone’s (2004) BSCS

BSCS items are prompted with this instruction: *Using the scale provided, please indicate how much each of the following statements reflects how you typically are.* Responses are a Likert scale consisting of 1 to 5 with the end points labeled Not-at-all and Very-much. The sum of the 13 items for each participant was computed, with reverse scoring as applicable. Thus, individual scores range from 13 to 65 with a neutral point of 32.5. Characteristics of the distribution of BSCS scores are shown in Table 4. The mean of 40.2 is greater than the neutral point and it is important to remember that this is a scale of “self-control” and that higher BSCS scores reflect more EF ability.

#### Whiteside and Lynam’s (2001) Impulsivity Scales

Three of the UPPS Impulsive-Behavior subscales developed by Whiteside and Lynam (2001) were included: (lack of) premeditation, urgency, and (lack of) perseverance. The urgency subscale consists of 12 items, for example, *When I am upset I often act without thinking.* The Likert-scale was *1 Strongly Disagree, 2 Disagree Some, 3 Agree Some, and 4 Strongly Agree.* High scorers on urgency are likely to engage in impulsive behaviors in order to alleviate negative emotions despite the long-term harmful consequences of those actions. The (lack of) premeditation subscale consists of 11 items, for example, *I usually think carefully before doing anything*. Low scorers are thoughtful and deliberative, whereas high scorers act on the spur of the moment and without regard for the consequences. The (lack of) perseverance facet has 10 items, for example, *I finish what I start*. Low scorers can remain focused on a task that may be boring or difficult. Characteristics of the distribution of these three impulsivity subscales are shown in Table 4.

#### Correlations Among the Self-report Measures of EF

The correlations between the five self-rated scales of cognitive control are shown in Table 5. All of the correlations are large except for those involving the premeditation subscale of impulsivity; however, those are still statistically significant. These scales appear to tap into the same general construct.

**Table 5:** Correlations between the five self-rated measures of self control

	Brief self control	Premeditation (lack of)	Urgency	Perseverance (lack of)
BDEFS	− .78**	+ .28**	+ .68**	+ .63**
Brief self control		− .35**	− .67**	− .71**
Premeditation (lack)			+ .24**	+ .27**
Urgency				+ .39**

*BDEFS* Barkley Deficits in Executive Functioning Scale

### Correlations Between Self-rated and Performance-Based Measures of EF

The five laboratory measures of EF were correlated with each of the five self-report measures of self-control. The full set of correlations are shown in Table 6. There is little support for the hypothesis that both sets are measuring the same general construct. No objective EF measure is significantly correlated with BDEFS, BSCS, or the premeditation subscale of impulsivity. In fact, all the Pearson correlations are less than r =|.09|. Two of the remaining 10 correlations showed small, but significant correlations. Specifically, perseverance, a subscale of the Impulsivity measure, and target-present slope in visual search were correlated, r(197) =  − 0.163, p = 0.022, thereby indicating as lack of perseverance increases visual search efficiency increases. Urgency, another subscale of the Impulsivity scale, is correlated with the spatial Stroop effect, r(195) =  + 0.183, p = 0.010, revealing an association between greater urgency and greater interference in the spatial Stroop task. Given that this is a set of 25 correlations and these bivariate correlations were not corrected for multiple tests, one might argue that there is very little evidence that self-control scales and laboratory measures of EF are measuring the same construct. This is consistent with the results and conclusions of Paap et al. (2020a), Toplak et al. (2013), and Duckworth and Kern (2011).

**Table 6:** Correlations between the five self-rated measures of EF and the five performance-based measures

	Switching costs	Mixing costs	Target present slope	Target absent slope	Spatial Stroop effect
BDEFS	− .005	+ .008	− .069	− .083	+ .069
Brief Self Control	.000	+ .036	+ .049	− .006	− .079
Premeditation (lack)	+ .016	+ .117	+ .077	+ .057	− .063
Urgency	+ .038	+ .030	− .029	+ .051	+ .183*
Perseverance (lack)	+ .014	+ .093	+ .163*	+ .115	− .074

*BDEFS* Barkley Deficits in Executive Functioning Scale

*p < .05

### Correlations Between Self-rated Measures of Self Control and AQ

To determine shared variance between AQ and self-rating measures of EF, AQ scores were correlated with each of the five self-reported measures (see Table 7). In contrast to the performance-based measures of EF, the relationships between AQ and self-control are quite consistent. To review, higher scores reflect better self-control for BSCS, but less self-control for BDEFS and the impulsivity subscales. Thus, higher AQ scores are associated with less self-control and this is not surprising under the assumption that AQ scores represent a continuum and that autism involves deficits in self-control. Our results showing that self-rating scales of EF consistently show negative correlations with AQ scores, but that the laboratory tasks were inconsistent at best, matches the pattern found in Demetriou et al.’s meta-analysis of studies examining the relationship between EF and diagnosed ASD. Thus, autism traits are related to deficits in EF, but the relationship is stronger and more consistent if EF is measured by subjective ratings of behaviors in everyday life.

**Table 7:** Bivariate correlations between AQ Likert scores and self-report measures of self control

	BDEFS	BSCS	Premeditation	Urgency	Perseverance
Pearson r	+ .424	− .243	− .068	+ .351	− .260
Sig. (2-tailed)	< .001	.001	.341	< .001	< .000
N	200	200	200	200	200

Given that the largest correlation is with BDEFS, one can explore the relationship between BDEFS and the AQ subscales with the set of correlations shown in Table 8. Inspection of the pattern of significant correlations suggests that the shared variance between AQ and BDEFS is nested primarily in the AQ subscales for Social Skill, Attention Switching, and Communication. The relationships between AQ Imagination and BDEFS are weaker and those between AQ Attention to Detail and BDEFS are weak and negative. Said another way, the autism traits reflecting difficulties with social interaction, switching attention, and communication are related to self-reported problems in EF, but issues in imagination or attention to detail are not.

**Table 8:** Correlations between the BDEFS and the AQ subscales

AQ subscale	BDEFS subscales				
	Time Management	Organization/Problem Solving	Restraint/Inhibition	Motivation	Emotion Regulation
Social Skill	+ .25**	+ .40**	+ .21**	+ .21**	+ .24**
Attention Switching	+ .33**	+ .52**	+ .23**	+ .33**	+ .46**
Imagination	+ .00	+ .18*	+ .17*	+ .08	+ .12
Attention to Detail	− .21**	− .24**	− .17**	− .17**	− .08
Communication	+ .39**	+ .54**	+ .38**	+ .33**	+ .33**

*BDEFS* Barkley Deficits in Executive Functioning Scale

As shown in Table 8 the two strongest correlations involve the Organization/Problem Solving subscale from BDEFS as this subscale enjoys correlations greater than + 0.50 with the AQ Attention Switching subscale and the AQ Communication subscale. These two high correlations are driven, in part, by items that appear to tap into the same problem. For example, a reverse scored item from the AQ Attention Switching subscale (*If there is an interruption, I can switch back to what I was doing very quickly*) is similar to an item in the BDEFS Organization/Problem Solving subscale (*Easily distracted by irrelevant events or thoughts when I must concentrate on something*). Examples like these support the view that AQ and BDEFS share similar items because symptoms of autism are thought to overlap with symptoms of deficits in EF.

Perhaps a greater insight could be gained if a BDEFS subscale can predict overall AQ scores. To explore this possibility a step-wise regression used each of the BDEFS subscales as a predictor of AQ total scores. The final model included only two predictors (Organization/ Problem Solving and Time Management) that accounted for 28.4% of the variance in AQ scores, but the lion’s share (26.4%) accrues from the Organization/Problem Solving subscale. Furthermore, the standardize beta coefficient for Organization/Problem Solving was large and positive (β = 0.669) whereas that for Time Management was small (β =  − 0.209) and negative. Thus, to the degree that total AQ scores reflect more than the sum of its sub-scaled parts, one might adopt a working hypothesis that the EF deficits most central to autism are those captured by the Organization/Problem Solving subscale of the BDEFS.

### Correlations with Activities Associated with EF

Given the substantial relationship between self-report measures of cognitive control and AQ scores it would be interesting to know if AQ predicts participation in activities that are presumed to be associated with, and perhaps enhanced by EF. Correlations between AQ scores and eight measures of such activities are shown in Table 9.

**Table 9:** Bivariate correlations between AQ Likert scores and activities associated with EF

	Music frequency	Music training	L2 Pro	L2 Use	Video-gaming	Mind-fulness	Exercise	Team sports
Pearson r	− .204	− .175	− .041	+ .032	− .057	− .328	− .327	− .337
Sig. (2-tailed)	.004	.016	.565	.656	.425	< .001	< .001	< .001
N	198	187	199	200	198	198	198	198

*L2 Pro* foreign language proficiency, *L2 Use* percent use of second-most used language

AQ is unrelated to bilingualism as indexed by either the proficiency or amount of use of a second language. The self-rating scale was the same as that used by Paap and Greenberg (2013) and ranged from 0 (no exposure to a second language) to 7 (more fluent than a typical native speaker). Given autism tendencies toward deficits in social interaction and communication it is somewhat surprising that increasing AQ scores were not associated with lower L2 proficiency or use.

Each of the other experiences were assessed with single probe items (and five-point Likert scales) that have been used in the literature previously (Paap and Greenberg 2013; Paap et al. 2020a). Like bilingualism, the frequency of videogame play was also unrelated to the AQ scores. In contrast, AQ was significantly correlated with both the frequency of playing a musical instrument and the amount of music training. Students with more autism traits are less involved in performing music. Likewise, as AQ scores increased the frequency of practicing meditation or mindfulness decreased.

### Relationship Between AQ and Exercise

#### All Forms of Physical Activity Combined

**Fig. 2:**
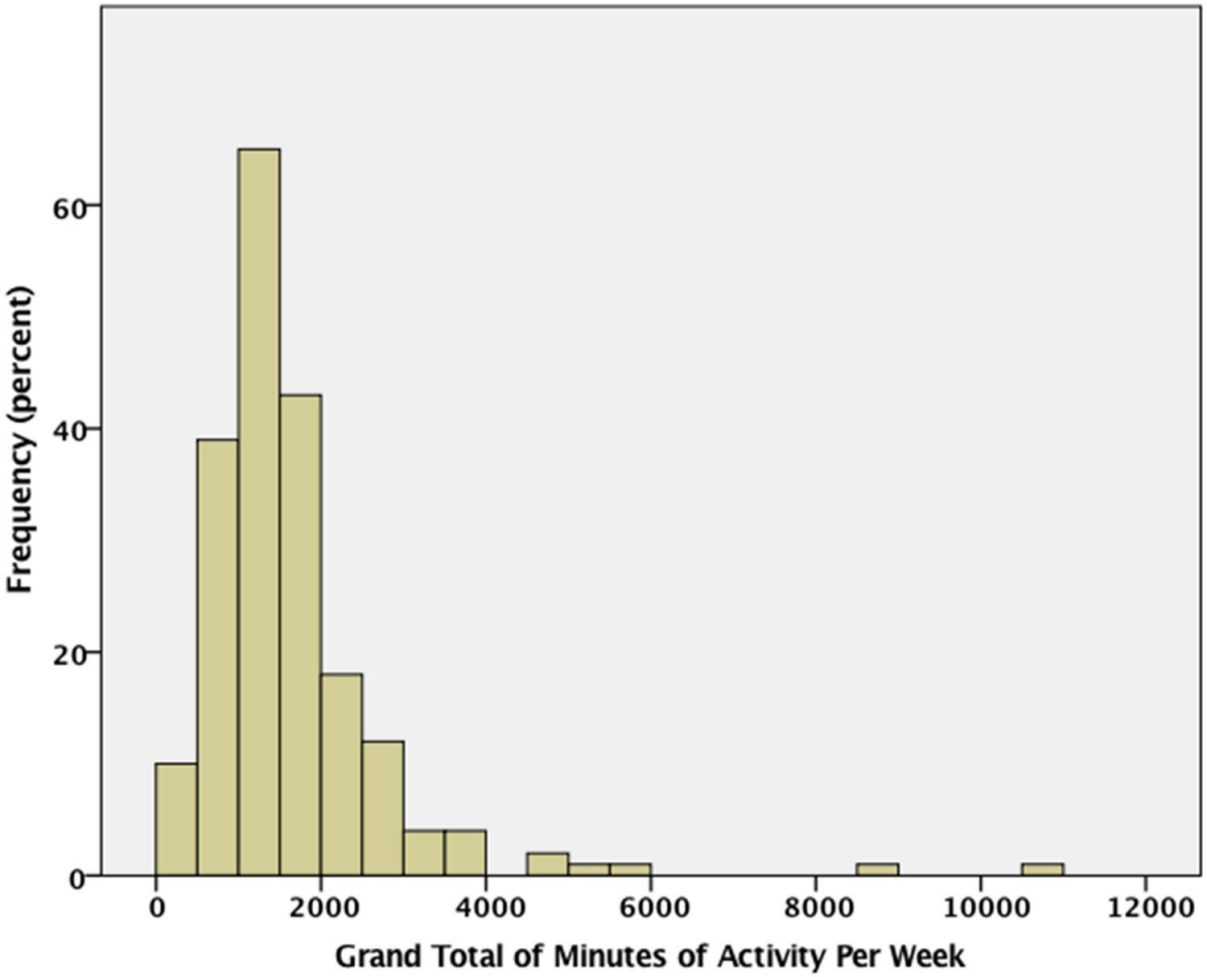
Percent histogram for grand totals (across all categories) of minutes of physical activity per week

We first explored the relationship between AQ and the grand totals across all types of physical activities and exercise types. Figure 2 is a histogram showing how participants differed with respect to the total number of minutes engaged in physical activity per week. Inspection of Fig. 2 shows that the distribution is positively skewed with most participants clustered near the mean of 1635 min, but with a string of super fit individuals scattered past 5000 min per week. One should note that these seemingly large totals also include low and moderately intense activities performed at home, at work, and walking.

**Fig. 3:**
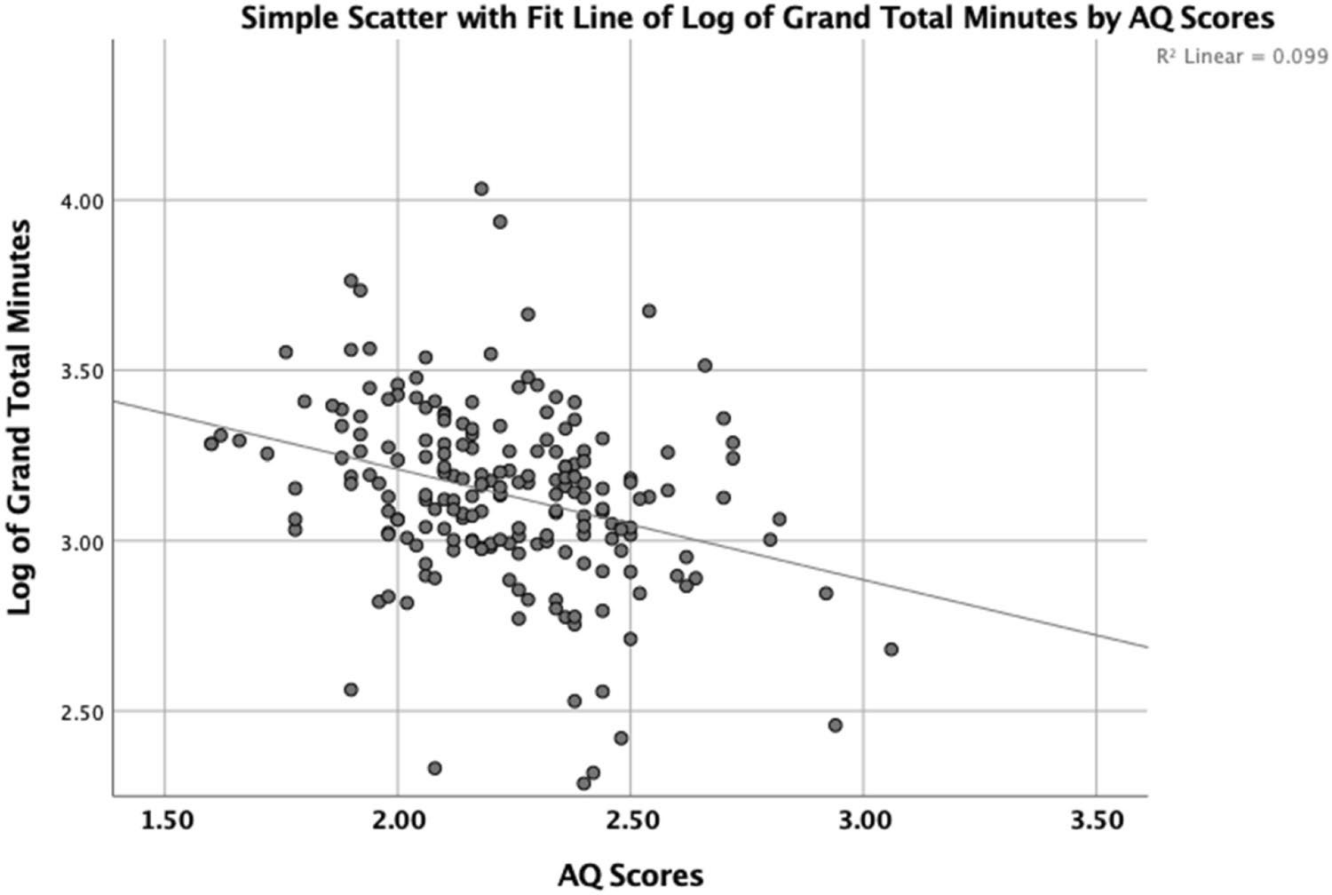
Scatterplot of log_10_ of the grand total number of minutes engage in physical activity per week as a function of AQ scores

Figure 3 shows the log_10_ of Total Time as a function of AQ score. The log transform compresses the positive skew and enhances the linear relationship between the variables. The correlation between the log_10_ of Total Time and AQ was significant, r(199) =  − 0.315, p < 0.001. The correlation with log_10_ of Total Energy and AQ was much the same, r(199) =  − 0.312, p < 0.001. Thus, in general higher AQ scores are associated with less physical activity.

#### Single-Person Exercise

Discretionary exercise is physical activity not associated with commuting, a requirement of your job, or routine house and yard work. Several sections of the CES cover different types of discretionary exercise. Perhaps the most fundamental category of single-person exercise consists of the nine activities shown in Table 10. Recall that for each activity we probe for frequency per week, duration per session, and intensity. The product of frequency and duration yields total time per week; and multiplying total time by intensity yields energy per week. The three base measures (frequency, duration, intensity) and the two composites (Total Time, Energy) can each be correlated with AQ scores and those are shown in Table 10 for the sub-category of Exercising. Generally speaking, each metric shows a significant correlation with the magnitude of physical activity decreasing as AQ increases. More specifically, each component correlates with AQ, but the composites are not better at predicting AQ; in fact, those correlations are numerically weaker. Comparing the three simple measures, it is noteworthy that greater autism traits are somewhat related to the frequency of single-person exercise, but more strongly and consistently related to the duration and intensity of the exercise.

**Table 10:** Bivariate correlations between AQ Likert scores and the 5 measures of single-person exercise

	Percent Participation	Frequency	Duration	Intensity	Total Time	Energy
Running	84	− .12	− .06	− .23**	− .04	− .04
Exercising (with weights, machines, or on the floor)	83	− .20**	− .29**	− .30**	− .16*	− .15*
Swimming	54	− .10	− .30**	− .30**	− .14*	− .13
Biking	38	+ .00	− .10	− .10	+ .05	+ .03
Yoga/Tai Chi/Pilates	38	− .16*	− .21**	− .17*	− .19**	− .19**
Climbing	23	− .04	− .04	− .05	− .02	− .02
Skating	20	+ .02	− .00	− .07	− .06	− .08
Skiing/Snowboarding	15	− .08	− .12	− .15*	− .06	− .06
Surfing	10	− .17*	− .18*	− .23**	− .13	− .16*

**α < .01, *α < .05

With respect to the specific types of single-person exercise, Table 10 shows the nine activities in descending order of their popularity, that is the percentage of participants who indicated a frequency greater than zero. If a substantial number of participants indicate zero frequency (i.e., they never participate in the designated activity), and consequently have zero average duration and zero intensity, then this severely restricts the variability needed to support a strong correlation. In that regard, the significant correlation for surfing is most likely anomalous.

The background questionnaire included this item from our previous work. *How often in a typical week do your exercise, work out, or participate in a sport? never. rarely, sometimes, quite often, very often.* This item seems most related to the Exercising activity from Table 10 and indeed the correlations between the old item with Exercising are: r(200) =  + 0.683, p < 0.001 for frequency, r(200) =  + 0.446, p < 0.001, for duration, and r(200) =  + 0.418, p < 0.001 for intensity. For the present dataset the correlation between the old item and AQ scores is r(198) =  − 0.327, p < 0.001. Thus, the CES validates that our old item may be a good single-item probe when brevity is needed.

**Fig. 4:**
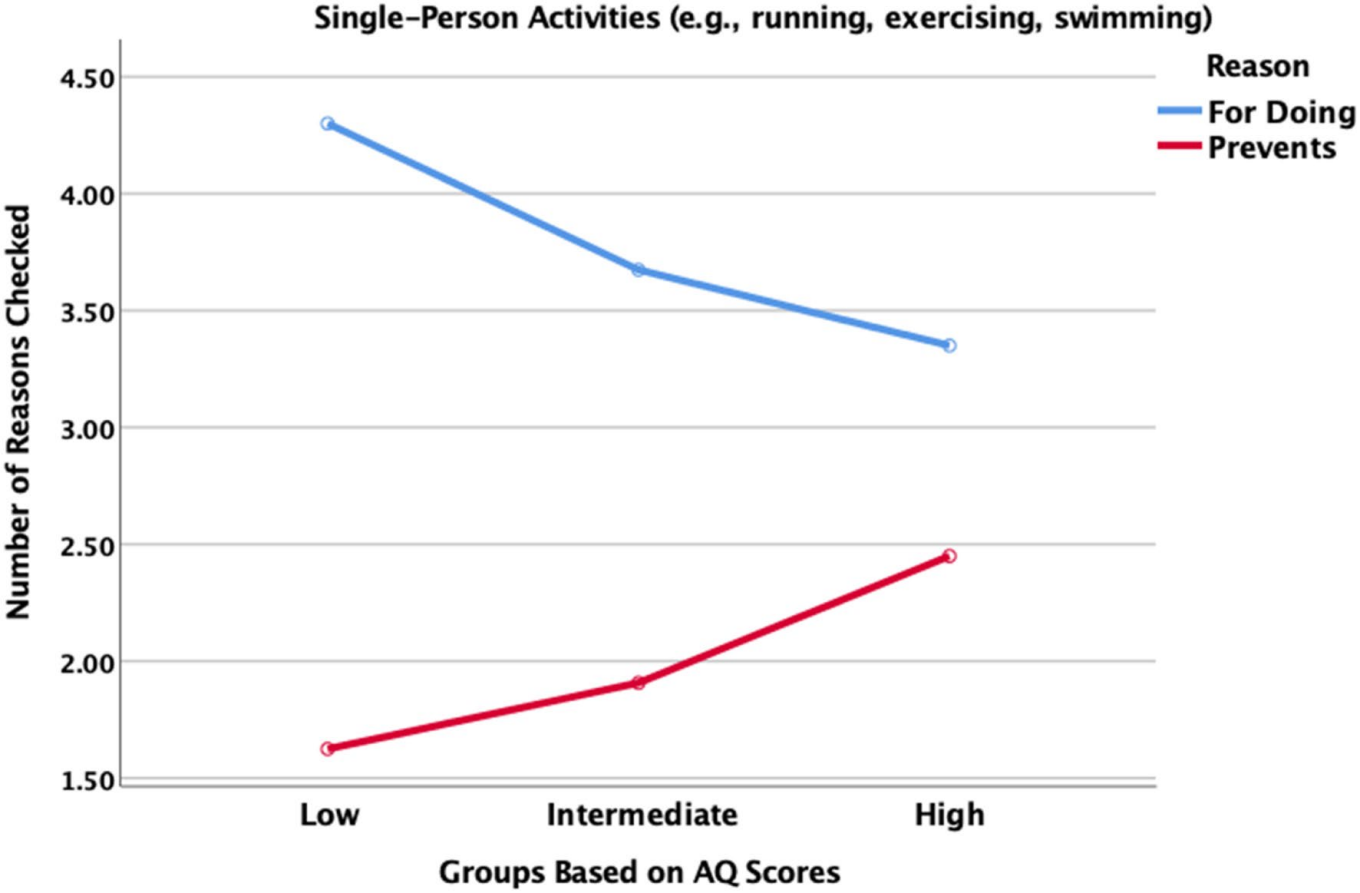
Number of reasons checked for doing single-person physical activities and number of reasons checked that prevent one from doing more of these type of activities

We were also interested in the reasons which motivated or prevented participants from engaging in single-person exercise. The number of reasons for motivation and prevention were tallied to determine a total score for both positive and negative reasons checked. Figure 1 shows the mean number for each condition formed by partitioning the AQ scores into three groups: Low (the low 40 AQ scores), Intermediate (the middle 141 scores), and High (the high 20 scores). This follows Stevenson and Hart’s recommendation to use three autism trait groups. A 3 × 2 mixed ANOVA yielded a significant Group × Type of Reason interaction, F(2, 198) = 4.15, p = 0.017, partial η^2^ = 0.040. The group with high AQ tendencies checks the fewest reasons why they are motivated to do the single-person exercise that they report doing, but the most reasons that prevent them from doing more. This suggests that increasing autism tendencies are associated with perceiving fewer benefits of exercise and greater costs (Fig. 4).

We also asked if participants preferred to do single-person activities alone, with others, or had no preference. As shown in Table 11, the group with high AQ scores did not show a systematic preference and their pattern of preference was identical to the large group with intermediate AQ scores. This supports a hypothesis that deficits or challenges with social communication are not contributing to the negative correlations between AQ scores and exercise. However, examination of the specific reasons given to justify their participation in single-person exercise suggest otherwise.

**Table 11:** Percentage of participants in each AQ group who prefer to do single-person physical activities alone, with others, or have no preference

	Prefer alone (%)	No preference (%)	With others (%)
Very high AQ scores	30	40	30
Intermediate	30	40	30
Very low AQ scores	15	59	26

Table 12 shows the percentage of participants in each AQ group who checked specific reasons that motivated them to do the physical activities they do (top panel) and the percentage who checked specific reasons that prevented them from doing more of these physical activities. It is important to note that the majority of those in the very high AQ group indicate that they exercise because it is good for their physical and mental health and that they do enjoy it. That said, there are potentially important differences in comparison to the other two groups starting with the contrast that a smaller percentage acknowledge the benefits to physical and mental health. Furthermore, and in contrast to the answers reported in Table 11, a smaller percentage of the high AQ group indicate that they enjoy the social experience and a larger percentage appreciate that they can do these activities alone.

**Table 12:** Percentage of participants in each AQ group who check a specific a reason that either motivates them (top panel) or prevents them (bottom panel) from engaging in single-person exercise

	AQ group		
	Very low AQ (%)	Intermediate AQ (%)	Very high AQ (%)
Reasons motivating single-person physical activity			
Good for my physical health	95	87.2	70.0
Good for my mental health	72.5	74.5	60.0
I enjoy it	82.5	66.7	60.0
I am good at it	40.0	22.7	25.0
Enjoy the social experience	40.0	31.9	15.0
Can do it alone	52.5	45.4	65.0
I have the time for it	47.5	39.0	40.0
Reasons preventing more single-person activity			
Makes me physically tired	30.0	38.3	50.0
Makes me mentally tired	7.5	11.3	40.0
Do not enjoy it	12.5	19.9	35.0
Lack skill or ability	12.5	23.4	30.0
Do not have the time	77.5	75.9	65.0
Have disability or condition	2.5	8.5	15.0
Costs too much	20.0	13.5	10.0

With respect to the reasons that prevent them from doing more of these physical activities, the most prevalent is lack of time; this is true across the range of AQ scores. This is not surprising given that most of the participants are full-time students and a substantial percentage also work. Perhaps the most striking contrast across the AQ groups was that the very high AQ group reports a higher incidence of the negative aftereffects of exercise making them feel physically and mentally tired, especially the latter. This is even more surprising if viewed in the context reported earlier that higher AQ scores are associated with shorter durations and lower levels of intensity. Finally, in comparison to the other two groups, the very high AQ group is more likely to report that they do not enjoy these activities or lack the skill or ability. These reasons may resonate with each other.

#### Team Sports Exercise

Participation in team sports is likely to be related to AQ scores because team sports place a premium on EF, social interaction, and communication. Paap and Greenberg (2013) introduced this probe: “Team sports often involve dividing your attention between a ball, a goal, your opponents, and your teammates. Do you excel at these sports?” In a series of regression analyses they reported that it predicted three measures of EF: the flanker effect (inhibitory control), switching costs, and flanker Global RT (monitoring). The significant regression coefficients (betas) were − 0.21 or − 0.22. This item is not part of the CES but was included in the general background questionnaire. For this dataset the team sports ability item did not significantly correlate with any of the laboratory measures of EF, but it did significantly correlate with the self-rated scales of cognitive control: *r*(198) =  − 0.221, *p* = 0.002 for BDEFS and *r*(198) =  − 0.183, *p* = 0.010 for BSCS. More important for present purposes, the team-sports ability item significantly correlates with AQ scores in the current dataset, *r*(198) =  − 0.337, *p* < 0.001.

Table 13 shows the simple measures of frequency, duration, and intensity and the composite measures of total time and energy for eight team sports included in the CES. As shown in the percent participation column, the majority of our student participants do not or no longer play team sports. This will limit the capacity of these measures to predict AQ scores. Nonetheless, AQ is negatively correlated with participation in football, baseball, hockey, and basketball. Consistent with the patterns observed for single-person activities, correlations tend to be stronger for duration and intensity compared to frequency, total time, or energy. Once again, the most striking contrast between the groups with respect to specific reasons (see Table 14) is the fairly substantial proportion (40%) of very high AQ individuals who indicate that playing team sports leaves them mentally tired.

**Table 13:** Bivariate correlations between AQ Likert scores and the 5 measures of team sports activity

	Percent Participation	Frequency	Duration	Intensity	Total Time	Energy
Basketball	37	− .09	− .13	− .18*	− .06	− .06
Soccer	32	+ .10	− .12	− .12	+ .09	+ .09
Volleyball	28	− .02	− .07	− .12	+ .01	− .00
Football	15	− .08	− .23**	− .33**	− .07	− .07
Baseball, softball, cricket	13	− .06	− .22**	− .22**	− .07	− .05
Hockey (ice, field, lacrosse)	3	− .16*	− .16*	− .18*	− .12	− .12
Water polo	3	+ .01	+ .01	+ .02	+ .08	+ .01
Rugby	1	− .04	− .11	− .13	− .10	− .10

**α < .01, *α < .05

**Table 14:** Percentage of participants in each AQ group who check a specific reason that either motivates them (top panel) or prevents them (bottom panel) from engaging in team sports

	Very low AQ (%)	Intermediate AQ (%)	Very high AQ (%)
Reasons motivating team sports			
Good for my physical health	70.0	87.2	87.1
Good for my mental health	60.0	74.5	72.6
I enjoy it	60.0	66.7	69.2
I am good at it	25.0	22.7	26.4
Enjoy the social experience	15.0	319	31.8
Can do it alone	65.0	45.4	48.8
I have the time for it	40.0	39.0	40.8
Reasons preventing more team sports			
Makes me physically tired	30.0	38.3	50.0
Makes me mentally tired	7.5	11.3	40.0
Do not enjoy it	12.5	19.9	35.0
Lack skill or ability	12.5	23.4	30.0
Do not have the time	77.5	75.0	65.0
Have disability or condition	8.5	8.5	15.0
Costs too much	13.5	13.5	10.0
Don’t have easy access to a team	40.0	50.4	42.5

**Fig. 5:**
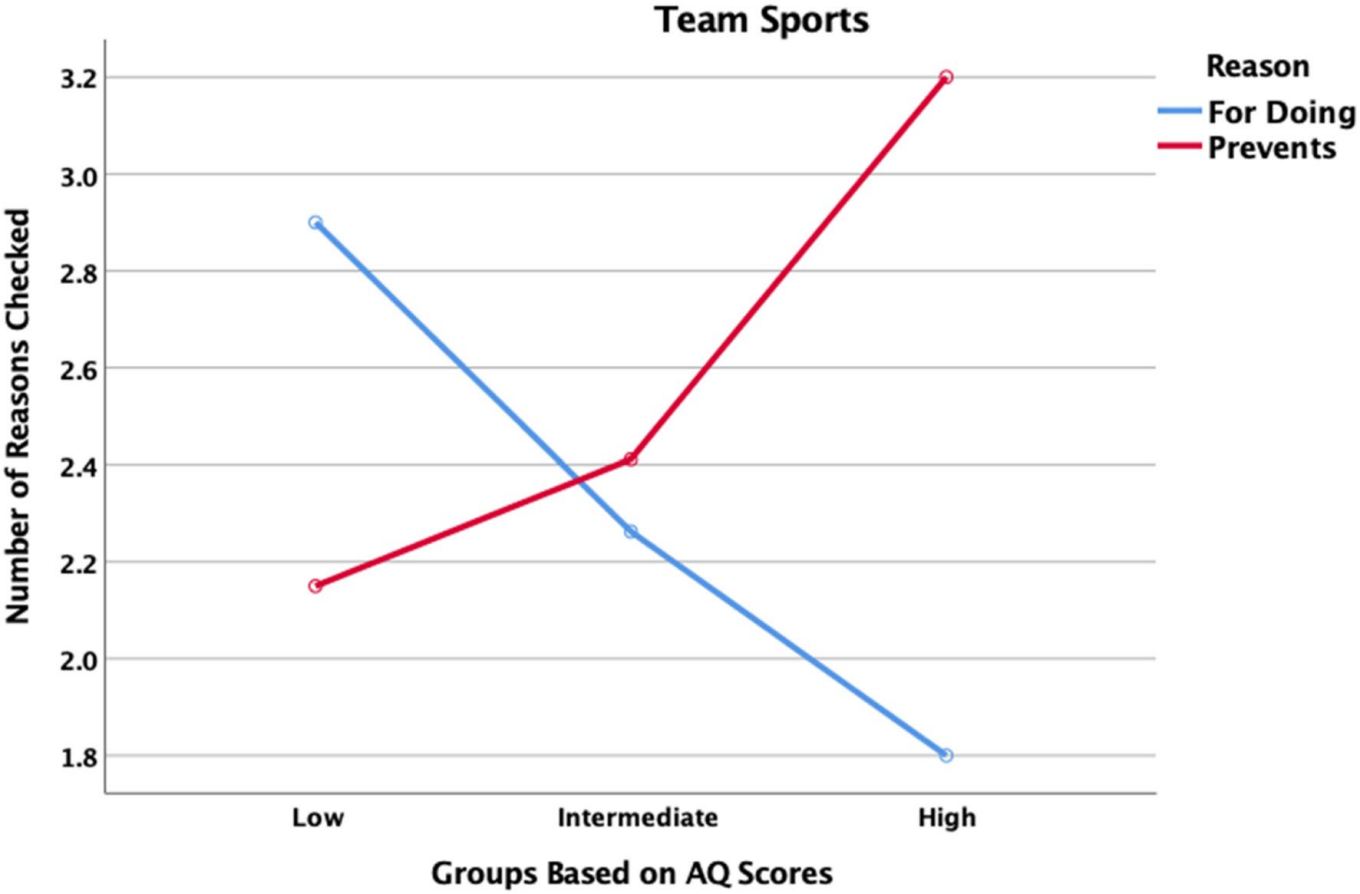
Number of reasons checked for playing team sports and number of reasons checked that prevent one from playing team sport

The total number of reasons motivating participation and the total number preventing participation in team sports was tallied for each participant and the mean number of reasons of each type for each of the three AQ groups are shown in Fig. 5. Again, there is a significant Group × Reason Type interaction, F(2, 198) = 3.745, p = 0.025, partial η^2^ = 0.036. The pattern is the same as for the single-person activities: as AQ tendencies increase, fewer positive reasons are checked, but more reasons preventing participation are checked.

#### Single- or Two-Person Ball Sports

Of the five sports shown in Table 15, bowling was the only one that the majority of our sample did at least a few times per year, although 42% played table tennis. As shown in Table 15 the frequency of play never significantly correlated with AQ scores for any of these sports, but duration did for bowling and table tennis and the largest negative correlation was observed between table-tennis intensity and AQ scores. In brief summary, these sports follow the trend that the duration and intensity of play decrease as AQ scores increase, but the strength of the relationships tend to be weaker than in other categories of activity.

**Table 15:** Bivariate correlations between AQ Likert scores and the 5 measures for each of the five ball sports usually played with one or two persons

	Percent Participation	Frequency	Duration	Intensity	Total Time	Energy
Bowling	61	− .04	− .21**	− .06	− .07	− .02
Table Tennis (ping pong)	42	− .11	− .20**	− .37**	− .09	− .09
Tennis	32	− .06	− .08	+ .03	− .08	− .09
Golf	16	− .01	− .05	+ .28	− .02	− .01
Racquetball, squash, handball	12	+ .02	− .06	− .21	− .03	− .05

**α < .01, *α < .05

#### Combat/Contact Sports

The CES treats boxing (12%), wrestling (6%) and any form of martial arts (8%) as a separate category. As indicated parenthetically only a small percentage of our sample participated in these sports and, consequently, it is not surprising that the AQ scores did not significantly correlate with any measure for any of the three sports.

#### Dancing, Walking, Climbing Stairs

In contrast, 64% of the sample dances and as shown in Table 16 significant negative correlations with AQ were obtained for the measures of frequency, duration, total time, and energy. In contrast to many other activities the correlation between AQ scores and intensity was near zero. For this set of activities, total time is the best predictor of AQ scores.

**Table 16:** Bivariate correlations between AQ Likert scores and multiple measures of dancing, walking, and walking-up stairs

	Percent Participation	Frequency	Duration	Intensity	Total Time	Energy
Dancing	64	− .17*	− .19*	− .02	− .20**	− .17*
Walking to school and job	100	NA	NA	− .07	− .26**	− .21*
Walking up stairs	100	NA	NA	− .02	− .20**	− .16*

*NA* not applicable

**α < .01, *α < .05

## General Discussion

How are autism tendencies (AQ scores) related to executive functioning (EF)? To answer this question we examined the relationship between AQ scores and: (a) 5 self-rating scales of EF, (b) 5 performance-based measures of EF, and (c) 5 types of activities or experiences (exercise, music, mindfulness, bilingualism, videogaming) that are assumed to recruit EF and sometimes enhance EF.

### Self-ratings of EF Predict AQ Scores

Before looking at the relationship between EF and AQ, it is important to show that the multiple measures of EF demonstrate convergent validity by correlating with one another. Table 5 shows that this is the case. The correlation between the two popular measures of self-control (BSCS) and executive dysfunction (BDEFS) was strong (r =  − 0.78). The correlations of BSCS and BDEFS with the three impulsivity scales were almost as strong for Urgency and lack of Perseverance. Self-ratings of control problems in everyday life can clearly cohere into a latent variable that likely captures a single psychological construct.

As shown in Table 7 there were significant correlations at p < 0.001 between AQ and four of the self-rating measures of EF. Not surprisingly, it is the lack of Premeditation scale that showed no association. According to Cohen’s (1988) guidelines, the four significant correlations have effect sizes between small and medium. These results merit the conclusion that as AQ tendencies increase, self-ratings of EF decrease. This generally supports the executive dysfunction hypothesis. Furthermore, it shows that the dysfunction is not a problem that impacts only diagnosed individuals, but rather it reveals itself across a spectrum of neurotypical university students. That said, the results do not speak to the question of whether there is a broad and general deficit in EF among those who scored high in autism traits. In fact, when stepwise regression analyses were used to determine which BDEFS subscales were driving the association, the results supported the working hypothesis that the Organization/Problem Solving subscale of the BDEFS may be a primary driver of the relationship between EF deficits and ASD. More research is needed to determine if the deficits in EF that co-occur in ASD are restricted to specific domains of EF.

### Performance-Based Measures of EF Do Not Predict AQ Scores

#### Convergent Validity

Five performance-based measures of EF were derived from three different tasks. Only one cross-task correlation (see Table 3) was significant: that between the spatial Stroop effect and switching tasks (r =  + 0.184). Given that Stroop effects are purported to reflect inhibitory control and switch costs are frequently assumed to be caused, in part, by inhibition, this is consistent with some convergent validity between measures of inhibition. The significant correlations between the slopes for target-present and target-absent trials in the visual-search task and between switching and mixing costs in the color–shape task are both within-task correlations and it is likely that they would correlate even if they do not isolate the same component of EF. Although the current design did not include many tests of convergent validity between measures of the same component of EF, there is a distressing lack of convergent validity in the EF literature, especially for inhibitory control (Paap and Sawi 2014; Rey-Mermet et al. 2018; Paap et al. 2020a).

#### Correlations with AQ

In contrast to the correlations with self-report measures of EF, there were no significant correlations (see Table 2) between any of the performance-based measures of EF and AQ (see Table 2). Given the lack of convergent validity between the performance-based EF measures, this is not surprising. It is also consistent with the Demetriou et al. (2018) meta-analysis of 235 comparisons between groups diagnosed with autism and control groups in that the effect for performance-based measures of EF was only g = 0.48 compared to g = 1.84 for self-ratings (mostly based on BRIEF). The 50 studies reviewed by Stevenson and Hart showed that AQ scores often predict task performance, but only a half dozen of these studies used any of the tasks that dominate the cognitive control–control literature (e.g., flanker, Stroop, Simon, cued task switching, N-back, short-term-memory span, operation span, etc.) and these specific studies uniformly showed no differences between the ASD and control groups. For the most part, there is no compelling evidence for a relationship between laboratory performance-based measures of EF and ASD. In the absence of the studies using self-rating measures of EF, one might conclude that the executive dysfunction hypothesis was false. A caveat is that the entire set of accuracy-based tests included in the Demetriou et al.’s meta-analyses did yield an effect size of g = 0.48 which guidelines characterize as moderate in size. Having made this allowance, it is also fair to note that Demetriou et al. found that “Only a very limited number of measures achieved the criterion of clinical sensitivity….” and that “The majority of the measures reaching clinical sensitivity were based on the BFRIEF questionnaire” p. 4.

### One or Two EF Constructs?

Performance-based measures of EF do not correlate with each other, do not predict AQ, and do not separate those with ASD from controls. In contrast, self-rating measures of EF do all of these things. Although we have added to the clarity of this contrast between performance-based measures of EF and self-ratings, we are not the first to observe that they appear to be measuring different things. Toplak et al. (2013) concluded that the two types of measure assess different underlying mental constructs with performance-based measures reflecting the efficiency of cognitive abilities and self-rating reflecting success in goal pursuit. They suggest that the two types of measures “assess different aspects of cognitive and behavioral functioning that independently contribute to clinical problems…. Both modes of assessment are useful and valuable, but they provide different types of information in the context of clinical assessment” p. 140. Likewise, McAuley et al. (2010) conclude that the two types of measures assess different aspects of the same underlying construct with performance-based tasks assessing underlying skills whereas self-ratings assess the application of those skills in everyday life. Another interpretation offered by McAuley et al. is that performance-based measures lack ecological validity because testing occurs in environments that are designed to minimize distractions, maximize support, and provide a high degree of structure in the form of clear instructions and well-specified goals. Because these conditions seldom prevail in the wild of everyday life, the two types of measures “do not engage the same set of skills”, p. 502.

Paap et al. (2020a) make a similar point about the ecological validity of self-ratings versus performance-based measures. They observe that the laboratory tasks are very sensitive to the participant’s calibration of speed and accuracy, a skill that has little relevance to delaying gratification (urgency), planning before acting (premeditation), or having the grit to persist in the face of adversity (perseverance). Either implicitly or explicitly, the computerized EF tasks almost always encourage the participant to go as fast as possible without making more than an occasional error. The mechanisms needed to filter out competing information in the nick of time, and when there is little intrinsic value associated with a “correct” response, may be different from those needed to resist actions that are affect laden and/or creatures of habit and have genuine costs and benefits. Moreover, competing information in the real world does not typically appear at random, it is not typically tied to the onset of new task relevant information, and the conflict need not be resolved within the first couple of hundred milliseconds of the onset of the event. In fact, any rapid suppression of responses counter to long-term goals often needs to be sustained in order to be ultimately successful.

All of these research groups agree to the general point that these two types of measures do not measure the same thing, but it may be time to consider whether the performance-based measures add any value to clinical practice. There is now an interesting and intense debate within the cognitive-control community regarding the reality of EF as a psychological construct. Performance-based measures of the same “objective” component of EF often show poor test–retest reliability and convergent validity (Paap and Sawi 2014, 2016; Paap et al. 2019). This problem is most acute for inhibitory control. Not only do two of the most common measures of interference control, Simon effects and flanker effects, not correlate; but different versions of the same task have near zero correlations. For example, the arrow versus letter version of the flanker task (Salthouse 2010) and four different versions of the Stroop task (Shilling et al. 2002) do not correlate. Comparisons of an ASD and control group typically use just one measure of inhibitory control. Inconsistencies across studies could be due to heterogeneity across ASD groups or to the choice of measure. Furthermore, even if one measure consistently produced disadvantages for the ASD group, it may be due to a task-specific mechanism rather than a general inhibitory control ability. In fact, one large-scale latent-variable analysis using 11 established tasks concluded that we should “stop thinking about inhibition” as a domain-general ability (Rey-Mermet et al. 2018).

In another large-scale study, Rey-Mermet et al. (2019) intended to examine the relations between latent variables of EF, general fluid intelligence (gF), and WMC. However, they could not establish a coherent latent variable for EF despite good reliabilities for all seven tasks. Even considered separately, none of the EF measures was found to be related to the gF or WMC latent variables. They conclude that the laboratory measures are highly task-specific and that the cumulative results question the seminal EF model proposed by Miyake et al. (2000) and its updates. Returning to present purposes there is, in our view, no compelling reason for practitioners to use these performance-based tests or to place much weight on research that either shows or does not show (most likely) a relationship between ASD and these purported measures of EF.

### The Unfortunate Consequences of the Failure of Performance-Based Measures to Cohere as a Latent Variable

This pattern of interaction (i.e., self-ratings of EF deficits correlate with AQ, but objective measures of performance on EF tasks do not) is disconcerting because correlations with objective performance measures carry greater causal implications. Consider what did not happen. Suppose that switch costs in the color–shape task significantly correlated with AQ scores. This would directly tie higher AQ scores with poorer performance in switching from one task to another. That is, AQ tendencies based on self-ratings would predict actual task performance when switching from one task to another. The AQ and objective EF measures derive from distinctive mental processes. Self-ratings of AQ items involve integrating episodic memories of similar situations and the resulting behaviors and then evaluating the frequency of classes of outcomes. In contrast, the switching task involves comparing RT when a switch is required compared to when the task repeats. The measure is in the moment (many trials over a span of 10 min). A correlation between AQ scores and switch costs would not prove that switching deficits are causing certain autism symptoms, but the evidence would be fairly compelling. It might, for example, motivate a training study that examines if adaptive practice in task switching leads to a reduction in AQ symptoms.

In contrast, consider what did happen: self-ratings of deficits in EF correlated with AQ scores. The processing underlying these measures are similar, not different. Both self-ratings of EF and the self-ratings in the AQ scale involve retrieving episodic memories of events similar to the one described in a test item and then evaluating the frequency of different outcomes. The significant correlation reinforces that individual experiences of autism traits and failures in EF tend to align, but the possibility that the latter is causal seems far less compelling. Adaptive training on taking the BDEFS is not likely to lead to lower AQ scores and, if it did, one might suspect that the intervention simply trains individuals on how to respond without necessarily changing behaviors in everyday life. To summarize the take-home point, the correlation between self-report measures of EF and AQ scores may modestly contribute to our understanding of ASD, but at another level it is simply explaining the symptoms of ASD by pointing out the symptoms it shares with deficits in EF.

### What Important Factors Do AQ Scores Predict?

AQ scores showed several interesting associations with activities that may recruit and enhance EF. College students with greater autism tendencies engaged in less exercise and rated themselves lower in team-sports ability. This negative relationship, together with the reasons high AQ scorers use to rationalize not exercising more, could lead to more effective interventions for improving physical fitness. While the preexisting literature on autism supports the notion that exercise interventions are efficacious in improving cognitive functions among those with ASD, as summarized by Tan et al. (2016), little has been known to this point about the underlying reasons individuals with autism engage in less exercise than their neurotypical peers. Our findings contribute to the literature on autism by bringing some of these subjective hesitations to light.

Likewise, students with greater autism tendencies practice less meditation (mindfulness). Although the correlations are not as strong, students with greater autism tendencies have also received less musical training and play instruments less frequently. At the AQ subscale level, these correlations are driven by the Social Skills, Attention Switching, and Communication subscales. As usual, one cannot draw causal conclusions from correlations, but a plausible hypothesis is that AQ tendencies could cause one to avoid activities that require EF and this, in turn, dampens the further development of EF. Further research may consider alternative interventions to optimize cognitive performance. However, it would be fair to consider that twin studies show that EF is highly heritable (Friedman et al. 2008; Paap 2018) and that this likely imposes limits on how much any type of EF training can improve symptoms of ASD, which are also highly heritable.

### Limitations

The present study was clearly limited in that we were not able to test a group of participants diagnosed with ASD to see if their outcomes were similar to those with high AQ scores. Despite the fact that we carefully chose five measures of EF that minimized known problems with reliability and validity, the failures to observe significant correlations between these performance-based EF measures and either the self-rating measures of EF or the AQ scores is another limitation of the study. It may be that performance on these tasks is mostly task specific and reflects very little about individual differences in general EF ability. However, Draheim et al. (2019) developed a battery of modified and new procedures for measuring EF that are accuracy-based and were shown to be more reliable and critically to have stronger intercorrelations than standard measures such as the Stroop, flanker, and others that rely on RT difference scores. This battery could be used in a future study.

## Conclusion

Self-ratings of executive dysfunction predict AQ scores. Performance-based measures of EF do not. Although some researchers believe that performance-based measures map onto a different, but useful facet of cognitive ability, we suggest that they may not. Nevertheless, the consistent finding that self-rated EF correlates with increasing AQ scores lends support to the hypothesis that autism often involves executive dysfunction and that this relationship holds across a wide spectrum of autism traits. New evidence was provided showing that AQ scores are negatively correlated with playing music, meditation/mindfulness, and especially physical activity. As autism traits increase there is a tendency to acknowledge fewer benefits of exercise and to perceive more costs. Interventions that increase the frequency and intensity of physical exercise might have the potential to generate considerable benefit among those with ASD. Finally, mindful consideration of the self-reported reasons for reluctance to exercise as a function of increasing autism traits may lead to more successful physical activity interventions in the future.

## Footnotes

1 Hedge’s g is a measure of effect size that expresses the difference of the means in units of the pooled standard deviation. Cohen’s (1988) guidelines suggest that effect sizes of about .2, .5, and .8 can be considered small, medium, and large.

2 These included shifting, mental flexibility, spatial cuing, visual search, finding embedded figures, global versus local processing, identifying morphing figures, object discrimination, hemispheric lateralization, saccadic suppression, humor preferences, anxiety, depression, the selection of prosocial actions, responses to facial expression, social learning, and social acting.

3 Some researchers using Likert scoring do not take the mean and consequently, individual scores range from 50 to 200. This does not, of course, influence the obtained correlations.
